# The NOTECHS+: A Short Scale Designed for Assessing the Non-technical Skills (and more) in the Aviation and the Emergency Personnel

**DOI:** 10.3389/fpsyg.2019.00902

**Published:** 2019-05-07

**Authors:** Andrea Ceschi, Arianna Costantini, Vivian Zagarese, Eleonora Avi, Riccardo Sartori

**Affiliations:** ^1^Human Sciences, University of Verona, Verona, Italy; ^2^Virginia Tech, Blacksburg, VA, United States; ^3^Helicopters Italia SRL Unipersonale, Trento, Italy

**Keywords:** NOTECHS, resilience, emotion regulation, HEMS, emergency, aviation

## Abstract

This research presents the development of a short scale named “NOTECHS+” to measure the Non-Technical Skills (i.e., NOTECHS: Cooperation, Leadership and Managerial skills, Decision-Making, and Situational Awareness), Resilience and Emotion Regulation, in a sector that comprises the aviation and the emergency personnel: the Helicopter Emergency Medical Service (HEMS). The design process of the scale was carried out starting from a review on the behavioral markers used to detect the NOTECHS. Moreover, 70 interviews with HEMS experts have been conducted with the aim of developing Resilience and Emotion Regulation items by considering the different professional profiles (e.g., pilots, nurses, physicians, etc.) which compose the HEMS. Through a pre-assessment procedure, a Q-Sort test was performed on a sample of students (*n* = 30) to test the logical principles, but also intelligibility and clarity, of the items developed. Once the instrument was defined, 211 participants from the HEMS sector were surveyed to test the theoretical model behind the NOTECHS+ instrument. First exploratory and then confirmatory analysis yielded results that suggested that the 18 items selected conform to a bi-factor model composed of three skill-dimensions: Social skills (i.e., Cooperation, and Leadership and Managerial skills), Cognitive skills (i.e., Decision-Making and Situational Awareness) and Emotional skills (i.e., Resilience and Emotional Regulation). Finally, the study ends with a discussion on the results obtained, including practical implications on assessment and training based on this novel instrument.

## Introduction

Aviation-related industries are to be considered high-risk organizations where responsibilities and pressures are high, as well as the physical and psychological risks related to the incidence and probability of accidents (Dekker, 2010). The operative core of such organizations is represented by flight crews, which are usually small-sized command and control teams, in a certain way unique, since they are extremely exposed to both high-risk factors and a high degree of human interaction (Salas et al., 2001). For such reason, a correct risk identification and an accurate accident analysis based on human interaction are fundamental to manage the safety levels of these teams. Moreover, this is the basis of the HR aviation culture where—given the nature of complex systems, characterized by human-machine interaction but also by teams' human-human interaction—human error represents the cause of a considerable percentage of accidents (Flin et al., 2002; Shappell et al., 2017).

Another aviation-related field, also extremely exposed to human errors, is the emergency sector, such as the HEMS (Helicopter Emergency Medical Service) field. The Helicopter Emergency Medical Service is an exceptional example of encapsulation of the medical and aviation sectors, since professionals belonging to both these fields (e.g., physicians, pilots, flight technicians, etc.) are working together and undergo various kinds of pressures (e.g., time pressure, peer pressure, self-induced pressures etc.) that could lead to human error. When carrying out HEMS operations, usually team members have to make difficult decisions in a very short time, and the consequences of a wrong decision may be extremely grave. In this sector, Cooperation and Leadership skills are fundamental, since during emergency operations the cohesion of the team members can be strongly tested. Communication and teamwork skills—together with the ability to make good decisions—have been addressed in a multitude of studies in this sector. This has led to the development of an assessment system called: Non-Technical Skills (NOTECHS). This term was coined by Flin et al. (2003) to define those cognitive and social skills needed to carry out safe operations and thus are complementary to technical skills. Flin et al. (2010) describe the functional process of the NOTECHS system, based on a tool developed from behavioral, social, and cognitive markers specifically designed for the aviation sector. Over time, other versions of the same assessment system have been developed with the aim of meeting the needs of other fields, such as the medical and emergency fields.

The specific definition of the NOTECHS behavioral markers makes the instrument a very useful means to accurately assess for certain professional roles. However, the flight crew encompasses different profiles, especially in the HEMS sector, which includes both aviation and emergency personnel. This might be perceived as a limit, since comparative analyses between these different roles are not possible with a unique instrument. A second methodological limit is related to the fact that NOTECHS does not include a psychometric scale for a quick assessment, which is useful for training evaluation, and in a dynamic sector such as the one of helicopter emergency services. Finally, a theoretical consideration. The current NOTECHS structure does not include the assessment of other relevant non-technical competencies, as the coping strategies used to manage stressful situations and emotions, which play a fundamental role both for operators in the aviation sector and for emergency workers (Weick et al., 2008; Cicognani et al., 2009; Setti et al., 2016). Considering these issues as a possible development in the assessment field, the purpose of this study is to develop a short psychometric scale named “NOTECHS+,” which allows a quick measure of the NOTECHS and of some new skills. Beyond NOTECHS original constructs, the scale also takes into account other competences, named Emotional skills, such as Resilience and Emotional Regulation (i.e., respectively the capacity to recover from a stressful event and to manage the emotions experienced). Moreover, such a scale can be applicable to different profiles of the aviation sector, including pilots but also technicians and the emergency crews (e.g., nurses, mountain rescue operators, etc.).

To reach the previously mentioned purposes, we will start with a review on NOTECHS and the components related to the management of emotional stressors. The literature review is important not only to identify correctly the components analyzed and needed to define the items, but also to design those theoretical models that may be validated through the subsequent statistical analyses. For the analyses, we will use a mixed approach. First, the procedure will be essentially qualitative, since it will consist in a series of systematic interviews and of a Q-Sort test for the development of the items. Qualitative procedure is important for considering aspects of different professional profiles in both the aviation and emergency fields, which can emerge through interviews. After having developed the scale and having collected the first data, exploratory and confirmative analyses will be performed to retain the most reliable items and to validate the new theoretical structure of the NOTECHS+ model, since it considers the new Emotional skills component. The study ends with a discussion on the results obtained, including the practical implications on the emergency and aviation contexts on which it is applied.

## The Origins of the NOTECHS System

The project that led to the development of the NOTECHS system was carried out by four teams of psychologists from different countries belonging to different organizations: the Netherlands (NDR), Germany (DLR), France (IMASSA) and United Kingdom (University of Aberdeen). The project was intended to develop a system of behavioral markers thanks to a research funded by the Directorate-General for Mobility and Transport of the European Commission (Flin et al., 1998; Van Avermaete and Kruijsen, 1998; Flin and Martin, 2001). In addition to the NOTECHS consortium, the project involved further research organizations (SOFREAVIA, DERA) and three airline companies: British Airways, Airbus and Alitalia. The NOTECHS system provides a systematic approach to assess the Non-Technical Skills of pilots flying during their actual activities or in a flight simulator environment. The set of elements is based on theoretical models identified from a literature analysis and is compared with the KLM SHAPE and the NASA UT LLC (Helmreich et al., 1997) systems, in order to confirm that the essential elements envisaged in the international standards had been included. As to evaluate the usability, reliability and validity of the NOTECHS system as an assessment tool, five aviation research centers and four aviation commercial organizations were involved in the project. Flin et al. (2003) designed an experimental study that included: 105 examiners, selected from 14 European airlines, who were invited to assess the pilots' NOTECHS in different scenarios. The results showed that 80% of the instructors provided coherent scores and 88% of them were satisfied with the consistency of the method. On average, their score differed less than one point on the five-point scale established by the same experts that had developed the scenarios. This is considered an acceptable accuracy level since 98% of the instructors were satisfied with the NOTECHS system.

The structure of NOTECHS is designed considering relevant components in the work and the organizational domain. They are composed of four components, two social and two cognitive, which are: Cooperation, Leadership and managerial skills (or simply Leadership), Situational Awareness and Decision Making. In turn, these components are subdivided into elements that correspond to behavioral markers. The components and the elements of the NOTECHS system are measured on a five-point scale (“*very good, good, acceptable, poor, very poor*”). Besides, an overall evaluation “*acceptable/not acceptable”* is required. This structure was chosen after having analyzed other systems and upon the advice of subject matter experts such as examiners and instructors.

The first NOTECHS component is Cooperation, and is defined as the skill needed to work efficiently in a team for the same purpose. Cooperation comprises four elements: team building and maintaining, supporting others, conflict solving. The second social skill component concerns the role of Leadership, defined as the capacity of leading a group of people or an organization in a reliable way (Yukl, 2005). The behavioral markers of the NOTECHS related to Leadership are: the use of authority and assertiveness, maintaining standards, planning and coordinating, workload and resources management.

The other two components, which are defined as Cognitive Skills, are Decision-Making skills and Situational Awareness. In the psychology domain, Situational Awareness comprises the constructs of perception and attention, since it involves a continuous monitoring of the environment and of any changes in it (Lauria et al., 2019). It is a dynamic and multidimensional construct that includes the ability to anticipate critical events. The first element of this NOTECHS construct is the aircraft system awareness, which includes the active knowledge of the modes and status of the aircraft systems, as well as of the energetic status. The second element is the environmental awareness, i.e., active knowledge of the current and future aircraft position (e.g., weather, traffic, terrain, etc.). The last behavioral element of Situational Awareness is defined as the sense of time available and ability to look forward when considering future and contingent conditions (i.e., is the anticipatory awareness of time).

Decision-Making skills are the fourth NOTECHS construct and they are especially relevant for the personnel working in high-risk sectors, under high-stress conditions and pressures originating from the need to make quick decisions in a very short time. Elements that were included into the Decision-Making component are: problem definition, diagnosis and option generation, risk assessment, option choice, and outcome review.

## What NOTECHS Do Not Measure: the Emotional-Coping Strategies

As stated above, NOTECHS are formed by two social categories and two cognitive categories, which have four constructs each and several behavioral elements. However, recent studies pointed out the ever-increasing importance of the development of coping strategies for stressful events in the aviation sector. In an overview on NOTECHS system and its future development, Kodate et al. (2012) ask for a future integration of the Systems Theory (i.e., the transdisciplinary study of the abstract organization of phenomena) in line with the recent development of the resilience engineering research. Also, Saurin et al. (2013) suggest to implement the resilience construct into the NOTECHS assessment. Resilience engineering is defined as an emergent discipline that stresses the capacity of a system to safely adapt to changes by limiting the emotional-stressful factors when facing unexpected events (Kodate et al., 2012). It is considered as an emergent job-person characteristic, in which the attention moves beyond human error to the knowledge of the system's and people's ability to adapt to changes by anticipating, managing and recovering from changing conditions, while managing emotional stress.

A similar construct close to Resilience is the ability to regulate emotions experienced at work, especially in demanding situations. Emotion regulation consists in automatic or intentional strategies used to begin, maintain, change the emotions felt during tense events (Gross and Thompson, 2007). Research suggests that emotion management is one of the major predictors of safety in the workplace, especially in presence of various stressful conditions, as it happens in the aviation context (Sexton et al., 2000) and in the healthcare system (Buruck et al., 2016). Moreover, in an aviation sector particularly subject to emergencies, such as the HEMS sector, the identification of the coping strategies needed to manage emotions in critical conditions assume even a more relevant role. The results of a study carried out by Cicognani et al. (2009) on a sample of 764 emergency workers show the importance of using and recognizing coping strategies for individuals that work in extremely stressful conditions, such as the emergency context, as to avoid the development of burnout or even of a post-traumatic stress disorder (PTSD) after an incident.

## Conceptualization and Development Procedure of the NOTECHS+ Inventory

Hereinafter, we will describe the phases that led to the conceptualization and the development of the NOTECHS+ scale, for the assessment of both of the NOTECHS and the management of emotional stressors. The study has been approved by the University of Verona, Research Ethics Committee. The next procedures were approved in accordance with the National legislation and the University guidelines. All the subjects involved, participated in the study on a voluntary basis and gave their written informed consent for both interview and the next survey studies.

The first step consisted in an analysis of the scientific literature concerning the NOTECHS assessment methods (Flin et al., 2003). By using research data engines such as PsycINFO and PubMed, studies that involved NOTECHS assessment, and that could contribute to the development of the items of the questionnaire, were collected. We analyzed the main characteristics of the identified studies, namely: number of participants, number of citations (Scopus), authors, year of publication, abstract, method, instruments, results, discussion. For each study or scale taken into consideration, the items, categories, elements, selected behaviors and technical indexes including factor loadings and measures of internal coherence were examined (see Table 1).

**Table 1:** Review of NOTECHS behavioral markers studies (alphabetically ordered).

**References**	**Method**	**Instruments**	**Participants**	**Analyses and results**	**Discussion**
Nergård et al. (2011)	Two-stage research project. Stage 1: series of interviews to pilots to collect different formulations and variations about the perceptions of a qualified pilot; Stage 2: based on the interviews, a scale was developed as to assess these attitudes.	Stage 1: 15 group interviews; Stage 2: questionnaire on piloting skills developed on the basis of stage 1. With a 10-point Likert scale Factorial analysis and main component analysis.	Stage 1: 250 pilots, who received CRM training in 2 different Scandinavian airlines, were interviewed in groups between 1997 and 2007; Stage 2: 174 pilots from 2 Scandinavian airlines were administered the questionnaire. Eighty six Chief Pilots and 88 first officers	The qualitative data showed the pilots' perception that an essential part of a good pilot to be a good crew member. Cooperation and interaction are seen as essential aspects. Situational awareness, self-efficacy and self-esteem were stressed. This study showed that there are four desirable factors in pilots: “knowledge,” “flying skills,” “CRM,” and “self-awareness.”	The study identifies pilots' common perceptions of the desirable skills in order to develop an assessment tool from the pilots' point of view. A comparison between the results of stage 1 and 2 showed pilots' parallel perceptions on desirable NOTECHS. The results of stage 1 show that the pilot should have a personal awareness and be committed to the development of their competence areas. The factorial analysis of the quantitative data, instead, resulted in the identification of four factors within technical and NOTECHS.
Flin and Martin (2001)	The first study aimed at assessing the current vision of the behavioral markers systems of British airlines, both with and without experience related to these systems. The second study aimed at collecting evidence on the use of behavioral markers systems in international airlines with experience in this field. Analysis of the contents of the answers coming from interviews and questionnaires.	Semi-structured interviews (telephone or face to face) for the British sample of this study Questionnaire for the international sample of the second study.	The first study comprises a sample of 11 British commercial operators. The international study comprises a final sample of 14 airlines.	The analysis of the content of the interviews and questionnaire showed that a behavioral markers system is available for 5 British airlines out of 12 but in no case, it is used for assessment. Conversely, this system is present in the whole international sample, and it is used for assessment in 12 cases out of 14. There is a general need for training on behavioral markers for examiners and instructors. Besides, pilots generally have a positive vision of the behavioral markers systems.	The British sample revealed some problems in the introduction of behavioral markers that could indicate that they had little experience with these systems. Conversely, international participants showed that they had a wide range and use of behavioral markers. Less than half of the airlines use them during all the training and assessment phases, which suggested that they are still being developed.
Hörmann (2001)	Design and development of training videos and of a method to calculate the reference indexes needed to calibrate each scenario to the four NOTECHS categories. Assessors participated to a one-day standardized session, during which they were asked to fill in a questionnaire on the professional background and organization culture, plus a short version of the Flight Management Attitudes Questionnaire (FMAQ). They then received training in the NOTECHS, and in the afternoon they watch the eight videos related to different scenarios. They had to assess them by using a five-point scale. At the end of each session, they were administered an assessment questionnaire, and a debriefing was carried out at the end.	8 video-scenarios in English, which represented a wide range of behaviors in flight. Questionnaire FMAQ.	105 pilot instructors belong to 14 different European airlines, coming from the smallest and the largest companies according to the different cultural clusters.	The results were analyzed basing on the hypotheses of the cultural difference classified by Hofstede. - Individualism-collectivism (ID) - Power distance (PD) - Uncertainty-Avoidance (UA) The main results did not show that there is a national cultural perception. Main differences are among different airlines of the same nation and instructors with different levels of knowledge of the English language. Other differences were noted among Eastern European airlines.	The study on the cultural impact on cooperation in pilots' teams in the European JARTEL project indicates that the effects of national culture are less strong than the local culture and the single company culture. The hypothesis that the organizational culture would have had a stronger impact on NOTECHS assessment than national culture cannot be confirmed by the results of this study.
O'Connor et al. (2002)	Design and development of video-scenarios and a reference assessment method. One day session of 15 experiments. The 3-h Training sessions were followed by 3-h experimental sessions. Finally, an assessment questionnaire was administered, and an open discussion was carried out as a debriefing on the general feelings to obtain qualitative data for interpretation of the results.	8 video-scenarios (chosen among 15): each of them had a set of design references that corresponded to different levels of NTS for each behavioral category (5-point scale) that the pilots had to explain. Set of reference assessment.	A sample of 105 pilot instructors coming from 14 different European airlines.	Internal consistency of the assessment made by the participants seems to be quite high. However, absolute differences between the assessment of categories and elements show that the Decision-Making category has less coherence, even if it does not reflect on the consistency between categories and determinants (pass/fail). As far as reliability is concerned, there was a high level of agreement between participants and experts at a category level. Besides, participants tended not to use the evaluation that was not observed, even when the reference evaluation was not observed. For what concerns inter-rater reliability, the level is quite high at a category level. However, at a level of determinants (pass/fail), the agreement among pilots was either very high or very low. For what concerns acceptability, the questionnaire data show that the assessors are very satisfied and deem this system to be adequate for the crew's NTS assessment.	High internal consistency. Reliability of Decision-Making and Situational Awareness is difficult to assess carefully. Short trainings do not seem to be enough to assess some scenarios. Difference between the determinants (pass/fail) needs to be described more in detail. For what concerns category, agreement among rate is high, and variance in score distribution is about 80% less than the variance associated with casual answers. NOTECHS system provides a structure that allows evaluating a pilot's individual NTS.
Gontar and Hoermann (2015)	Examiners assessed pilots' performance in four video scenarios by using three different assessment tools The RWG for each dependent variable and the coefficient ICC of each measure was calculated by using a two-way model for each dependent variable.	Videotapes: four scenarios with four different crews operating in an A320 flight simulator. Two assessment tools for NTS: one based on items and the other one based on dimensions (Burger et al., 2003), LOSA (Line Operations Safety Audit).	37 assessors. Instructors with the wider experience came from a European airline and participated in a theoretical and practical training on assessment skills.	Results show that inter-group agreement and inter-rater validity cannot always be considered acceptable. Exceptional pilots' performance obtained the higher intergroup agreement. For what concerns performance cognitive aspects, inter-rated reliability showed to be higher than for social aspects.	Results show that reliability exercises are to be recommended in NOTECHS assessment training. Airlines and training organizations should be encouraged to demonstrate an adequate inter-rater reliability.
Flin et al. (2003)	Revision of existing systems for NOTECHS assessment as to identify common behaviors: research in literature of important outcomes concerning the main categories of NOTECHS identified in the existing systems discussions with experts, KLM pilots A system prototype was developed and discussed in a two-day workshop in which four team psychologists were involved. Components were revised during meetings with KLM pilots.	KLM WILSC/SHAPE systems (Antersijn and Verhoef, 1995) LLC system of Texas University (Helmreich et al., 1997); systems used by Air France; RLD (Dutch CAA) and Lufthansa (Quick Reference System).	For the JARTEL study, a consortium of 5 aviation research centers and 4 airlines was created to test the NOTECHS method.	The NOTECHS system includes four categories: *Co-operation, Leadership and Managerial Skills, Situation Awareness, Decision Making*, each of which is subdivided into elements, and behavioral markers.	The NOTECHS system resulted to be a practical professional tool for authorized instructors and examiners. This tool can be used by non-psychologists and uses the common professional aviation language. It intends to examine pilots and communicates concrete suggestions to improve. The preliminary assessment of the NOTECHS system coming from experimental and operation tests showed that its psychometric properties are acceptable and that the method results to be appropriate and accepted by professionals.
Thomas (2004)	Observation design. The observers collected data during normal operations. The observers received a two-day training on technical and logistic aspects of data collection. The results of crews' threat management were registered as a single dichotomic variable. Context factors and crews' NOTECHS were examined as a possible threat and error management predictors. Qualitative data were collected in the form of writing. Descriptive and multivariate analyses were performed.	A total of 323 observations were performed. The behavioral markers were adapted from NOTECHS and LOSA methods.	A group of 25 senior flight crews made up of a captain and a first officer, trained in the use of LOSA and belonging to a South-East Asian airline di working both in domestic and international flights.	During the 323 observations, observers found 508 errors. Crews did not notice almost half of the errors and less than a quarter of the total number of errors were managed efficiently. Results underline the importance of assertiveness in high-risk activities. Two categories of NOTECHS (situational awareness and decision-making) are significant predictors of an efficient crew's threat management during all the phases of flight.	The contribution of contextual factors and NOTECHS to threat and error management was assessed. Results provide empirical support to the importance of NOTECHS to minimize risk and improve performance. Besides, contextual factors are important for an efficient threat and error management. This kind of performance assessment may provide precious information to organizations to improve their training system by developing scenario-based training.
Prati et al. (2010)	The self-report questionnaire was uploaded on the University of Bologna. Multiple regression analysis was used to evaluate the effects of interaction.	Perceived Personal Efficacy for members of volunteer associations (Barbaranelli and Capanna, 2001), and the Italian version of Professional Quality of Life Scale Revision IV (ProQOL R-IV; Stamm, 2005; Prati et al., 2010). Scales on compassion satisfaction, burnout and compassion fatigue were included.	Sample of 451 Italian rescuers: firefighting members, Italian Civil Defense volunteers, and various medical professionals from the emergency sector.	The ration between stress evaluation and professional life quality was significant only for rescuers with low self-efficacy. Stress evaluation had a significant impact on compassion fatigue, burnout and compassion satisfaction, when self-efficacy values were lower. These results confirm the hypothesis that self-efficacy buffers the impact of stressful events on professional life quality.	The study confirmed the hypothesis that self-efficacy might have a “recharge” effect. It was shown that rescuers' belief that they had a certain control in stressful events promotes resilience. The extent to which rescuers feel that they can face the challenges coming from their activities may function as a self-fulfilling prophecy. Expectations regarding adaptation lead to resilience. The results suggest that interventions aimed at developing rescuers' psychosocial skills are useful.
Ruff-Stahl et al. (2016)	The test subjects were unexperienced pilots. They had to pilot two traffic models, assisted by an instructor pilot sitting in the right seat. The IP provided some flight instructions and then provided other verbal instructions during the assessment phase. Two independent assessors, sitting behind IP and SP, used the JARTEL markers and discussed the results after the flight. The assessment focused on SP's CRM skills.	Flight Trainer (FNPT2, simulating DA 42—TDI, Twin Star), 5-point Likert scale.	Unexperienced pilots (SP), instructor pilots (IP), two experienced assessors. *Verkehrspilotenschule training center in Berlin* provided a Flight Trainer (FNPT2, simulating DA 42 –TDI, Twin Star). Eight test subjects completed the experiment.	The individual category receiving the lowest score was Leadership and managerial skills, while the higher score was obtained in Decision-Making, followed by Cooperation. In general, categories were scored very differently by the different SP's and some categories were scored by a few or none SP. This is due to actual flight differences in the simulator.	The experiment shows the practical usage of the NOTECHS assessment grid for the selection of airline pilots. The results show that it is possible to evaluate unexperienced pilots, even if the system was designed to assess experienced pilots for requalification. The results are clear and unambiguous and show that NOTECHS is a tool that can also be used by non-psychologists. Variations in intersocial relationships between SP and IP (due to flight differences in the simulator) show that NOTECHS is useful in actual CRM scenarios. This pilot-study indicates that NOTECHS can be used for pilots selection purposes.
Brannick et al. (2002)	Based on a need analysis, instructors, and pilots were interviewed to develop a scenario for CRM evaluation. Instructors received a 3-day training session. The 2 pilot instructors watched 45 videotapes of the crews on a full-motion simulator and filled in in the observation grid. After that, they completed a checklist of the observed behaviors and a record card for specific, general behaviors and CRM assessment.	Two members formed the crew of the helicopter (TH-57): pilot in command and co-pilot. The scenario. Two record cards: one for the observation and one to record behaviors and evaluations.	The Navy made available for the study 50 full-motion flight simulator sessions. Pilots were coming from two different teams and were randomly assigned to the crews. The assessors were Navy TH-57 Pilot instructors. Three psychologists and three helicopters pilot instructors developed the modules.	Inter-rater correlation was higher for specific behaviors and evaluation of events than CRM items. For internal consistency, ANOVA was used to measure the effects of the type of item and rater, and no effect was found for the rater. Scales were prepared for the average score of the raters regarding specific behaviors and evaluation of events and each dimension of the CRM. The correlation matrix shows that all the scales correlate. Correlations among CRM dimensions indicate that for the instructors there is a little difference among the various dimensions.	The study was aimed at verifying the reliability of the instructor's evaluations of the crews' behavior during a simulated flight. Inter-rater reliability and internal consistency were good for the items than concerned the assessment of the crew's behaviors to the scenarios provided. CRM scales showed low inter-rater reliability. Besides, the scales on specific behaviors showed good inter-rater reliability but low internal consistency.

The markers considered in the various studies were then analyzed. Based on the similarity among them, we decided to take into account especially the markers from the study conducted by Flin et al. (2003) since they included all the constructs that could help develop the items of the scale. This procedure allows benefiting of all the various behavioral markers, subdivided into good and bad operational behaviors, for every single element of the four NOTECHS constructs. Some limitations related to the applicability of such assessment systems emerged. For instance, the study of Robertson et al. (2014) takes into consideration the same four NOTECHS components, but the examples of related operational behaviors resulted to be very specific for technical-assistant personnel. The instrument developed by Nergård et al. (2011), instead, refers mostly to the characteristics of the pilot, but does not consider other roles such as technicians. From all the scales presented in Table 1, we searched for all the possible behavioral markers that could define all the four NOTECHS skills, and that could be adapted to a larger segment of the aviation personnel and of emergency crews. First, we translated these practices into Italian, and we selected the markers that, according to us, could be adapted as items on the basis of their content. The following step was to turn them into a first-person sentence since the final questionnaire is a self-report instrument. After that, we maintained five items for each construct, since not all the items were applicable to all the operators, regardless of the role they played. For instance, as it concerns Leadership, the items “*I assign the tasks to the team members, I check and correct them in an appropriate way*” or “*I give the team the appropriate time to complete the tasks*” were discarded since they did not apply to operators that do not manage other people. Thus, our choices were made as to be exhaustive by taking into account the different roles in HEMS, reaching the overall number of 20 items (five for construct) for the classical NOTECHS categories considered.

### Critical Incident Interviews and the Development of Resilience and Emotion Regulation Items

Since NOTECHS literature does not include investigations on coping strategies for managing emotional stressors, we have carried out 70 interviews on an Italian sample of pilots, hoist operators, doctors, nurses and mountain rescue operators working in the HEMS sector. Sample recruitment was managed by a trained organizational psychologist with experience in the aviation and emergency sectors. The purpose was to understand the role of these variables in such a strategic sector, such as HEMS, since it comprises both aviation and emergency personnel. Focus-group, panels and interviews were carried out by using the *critical incident interview* method to explore an event that had actually happened during rescue operations, and that was also emotionally critical to the person interviewed. Then we focused on the individual actions taken to face the emotional stressful situation and its consequences. Below we provide some extracts from the interviews where, starting from the description of a critical incident, we investigated the coping strategies used by the individuals:

“*Our work is emotionally intense, even if I feel quite detached, everything is calculated–I re-elaborate things a little bit. Interventions on children are more stressful since we are parents as well – I do feel the responsibility*.”

“*When I am working, I do not feel strong emotions. We are not hard-hearted people, but we have to work. I have been doing this job for 30 years; you must concentrate on what you have to do, you do not have to be overtaken by emotions. When you get back to the base, you think of it, but we are lucky, since usually after one intervention we immediately have another one, so you do not think about it anymore*.”

“*Emotions always come late, when operations are concluded. In that very moment (when you work) you are clear-headed and concentrated. Pilot and technician are less in contact with emotionally stressful situations than the rest of the crew. The most important thing is to talk about it immediately. Emotions are not shared, only technical aspects are*”

Here are some statements of how the concept of resilience is perceived:

“*Once my head was more “behind my seat” (where doctor and nurse are sitting), while now I do not even see the face of the patient. This began after a rescue ended up very bad with a child. This thing works, I work better like this*”

“*We have a reduced RAM in which we try to keep only aeronautical issues, and we try to reset all the rest – as to start with a “white sheet*”.”

From what resulted from the interviews, due to the nature of this job environment, HEMS sector cannot be devoid of emotions and of recovery and resilience strategies used to face emergencies. From a resilience perspective, adaptation to emergency is an important element for coping with highly emotional and unexpected events. The capacity to adjust and adapt comprises knowledge in terms of anticipation (what to expect), attention (what to look for), response (what to do) and finally learning. This requires the ability to rebuild the personal resources quickly after a crisis and this process is fostered by emotional self-regulation capacity (Fredrickson and Losada, 2005). Recovery from emergencies is promoted by reappraisal and emotional self-regulation processes, by broadening one's thoughts and actions, and it is connected to the resilience level of the individual. Emotional self-regulation, and in particular cognitive reappraisal, which involves reframing a situation in order to change its emotional impact, helps in this situation, even if during the crisis the suppression of panic feeling might be more strategic (Mauss et al., 2007). Therefore, in order to develop the items, we considered the extracts from these interviews, along with some sample of items derived from the most reliable scales (based on citations and adaptability to the HEMS context) validated in Italian for assessing Resilience and Emotion regulation levels. Emotion regulation items derive partially from the cognitive reappraisal and emotional suppression subscales of the Emotion Regulation Questionnaire (ERQ) by Gross and John (2003). Example items are: “*When I want to feel less negative emotion (such as sadness or anger), I change what I'm thinking about*” and “*When I want to feel more positive.”* The Italian version of the Connor-Davidson Resilience scale (CD-RISC 25) has been considered for the development of the Resilience subscale (Connor and Davidson, 2003). Based on these samples of items and on the contents extrapolated from the interviews, we developed other ten items (five for construct) that were added to the 20 selected previously, as to assess a total number of six constructs.

### Development of the Items: Analysis of Intelligibility and Clarity, Q-Sort Test and Multi Correspondence Analysis (MCA)

To increase accuracy, we decided to perform a Q-sort test on a sample of students. The sample was composed of 30 Italian students of Psychology, the 70% of which was female. The sample average age was 28 with a standard deviation of 9.1. One third of the subjects already had a bachelor's degree, while three subjects a superior education qualification. We analyzed the levels of intelligibility and clarity of this set of items through a pilot-questionnaire. The Q-sort test is a research method used to investigate how people think with regard to an issue (inter-rater comparison). This method is used as a tool in assessment and it shows to be particularly useful when the researchers wish to understand and describe the variety of subjective points concerning the face validity of the instrument. The observer starts with the set of items (Q-set) for evaluating the psychological construct to which the items were referred. The observer shall decide the degree of similarity of each item with some possible typical behaviors. After having decided, he/she will order them basing on their similarity, classifying them into a number of pre-determined groups. In this case, we deemed it useful to use this instrument and therefore we prepared the necessary material. Each participant was given an envelope containing 30 cards—the Q-set - and six paper clips. They were asked to group the items in accordance with a principle of logic and similarity. We did not provide the names of the corresponding categories. At the end, we administered the questionnaire with the same set of items, in order to investigate the degree of intelligibility and clarity of every single item on a scale ranging from 0 (not at all) to 4 (very clear).

Before analyzing the Q-sort result, we first checked the average score of intelligibility and clarity of the various items, setting a cut-off value of 2.5. Thanks to the analysis on the degree of clarity, we found out that the item 12 “*I can recognize new voices, unexpected situations and changes in the instruments”* and item 26 “*When I want to feel less negative emotions I change the way I see things”* were critical since their average score was respectively 2.3 and 2.4. At the same time, thanks to the analysis on the degree of intelligibility, we found that item 7 and 26 were critical, since their average score was 2.4 and 2.3, respectively. Besides, the researcher in charge of the preparation of the scale and administration of the Q-sort and of the questionnaires reported that some participants showed to be puzzled when associating those items according to logical principles related more to the lexical structure of some items rather than to their content. For instance, the prosocial aspects of the Cooperation items could induce social desirability by leading the responder to acquiescence in responding (Ray and Pratt, 1979; Ray, 1983; Spector, 1992).

After that, a contingency matrix through the row and column profiles was created by using dichotomous variables in which rows correspond to the relative frequencies of the items for all the categories created by the students with the Q-Sort method. The multiple correspondence pattern showed that - with 73% of cumulative inertia - six categories could explain much of the variance. The general rules in MCA suggest that the number of categories retained should be higher of 70% of the inertia explained or correspond to the number right before the “elbow” of the eigenvalues by category number of scree plot (see Sourial et al., 2010). Multiple Correspondence Analysis is an exploratory descriptive method that uses the multivariate extension of the Correspondence Analysis for examining tables containing three or more variables. The present data analysis and the next ones were performed using the IBM SPSS 21.0 package, and the IBM AMOS 21.0 package (SPSS Inc., Chicago, IL) for the CFA analysis. Multiple Correspondence Analysis can be considered a generalization of PCA for categorical variables that reveal patterning in complex data sets without requiring an assumption of underlying normality (Ayele et al., 2015). Therefore, we deemed that six components were fully sufficient to define the six constructs (i.e., Cooperation, Leadership, Decision-Making, Situational Awareness, Resilience and Emotional Regulation).

### Revision of the Items and Definition of the NOTECHS+ Scale

In light of the items' distribution that emerged from the MCA resulting from the Q-sort and from the levels of intelligibility and clarity, we decided to revise the set of items to enhance their clarity. First, the items belonging to the Cooperation construct were turned into reverse score (items scale have been worded in the opposite direction), in reference to the poor practice detected by Flin et al. (2003). This was done with the objective to avoid response biases associated with social desirability effect (e.g., acquiescence, straight-line responding, etc.). Item 7 “*I succeed in motivating my crew thanks to recognition and I support the crew when necessary”*—which was critical from the intelligibility point of view—was modified, since it comprised two different concepts in one single sentence. Then, since Situational Awareness items were often associated with other categories, we replaced the term “situation” with the term “conditions,” as it has a more precise meaning compared to the previous one. Furthermore, item 15 “*I discuss time limitations and strategies in emergency situations*” was revised since the term “I discuss” at the beginning of the sentence diverted from the contents of situational awareness. Within the construct of Decision-Making, item 18 “*I tend to ask other crew members for new options before making a decision*” and item 20 “*I discuss the possible risks when making decisions, always considering crew limitations*” resulted to be critical from the MCA since they involved more categories. Therefore, these items were modified to address the decision made, rather than the contents of cooperation. For what concerns resilience, item 22 “*After being sick, after an accident or another serious situation I recover quickly*” and item 24 “*Even when I am under pressure I can keep concentrated*” were slightly modified, stressing the contents related to resilience rather than those that could be related to emotion regulation. Item 26 “*When I want to feel less negative emotions I change the way I see things*” was modified due to its low levels of clarity and intelligibility, perhaps due to the double negative.

In conclusion, the questionnaire to test is made up of 30 items referring to the six constructs and three dimensions: Social skills (i.e., Leadership and Cooperation), Cognitive skills (i.e., Situational Awareness and Decision-Making) and Emotional skills (i.e., Emotional regulation and Resilience). Answers to every single item are given on a 5-point Likert scale (“*I do not see myself in it at all; I do not see myself much in it; I see myself in it; I see myself a lot in it; I fully see myself in it”*).

## Analysis for the Scale Validation

After having defined the scale, we started the validation process by administering it to a sample of people working in the HEMS sector. The first goal was to test the reliability of the scale for each of the constructs. We considered the possibility to remove the items that were not reliable since we wished to create a reliable instrument, quick to administer and suitable to the dynamic context of HEMS. The second goal was to investigate the latent component associated by the instrument both in an Exploratory (PCA) and Confirmative (CFA). Since two new components had been added to the NOTECHS theoretical framework, we could expect that the latent factors could differ from the original NOTECHS configuration. For instance, we can expect a second latent factor which includes the extra components (i.e., Resilience and Emotional Regulation) added to the original NOTECHS, or three dimensions based on a skills-structure (i.e., Social skills, Cognitive skills, Emotional skills).

### Sample and Descriptive Statistics

We surveyed 10 Italian HEMS bases. We used a simple random sampling method to select the potential participants who were invited to take part to a survey aiming at studying NOTECHS components at work. Participation was voluntary. Once that participants agreed, they were informed about the focus of the survey and choose to fill a questionnaire online or using paper and pencil. We selected 225 participants, out of whom 14 did not complete entirely the questionnaire, and therefore they were excluded from the statistics, formed the sample (*N* = 211). The descriptive analyses regarding the professional roles show that most subjects are nurses (33.2%), followed by rescue operators (28.9%) and doctors (27.5%). The role of pilot (6.6%) and technicians are less frequent (3.8%). In the group of participants, the 79% were males and 21% female, while one subject did not specify his/her gender. Age was subdivided into clusters: three at 15-year intervals, the last being “more than 60 years.” From a first descriptive analysis, most participants of the sample belong to the cluster 31–45 years (48.8%), followed by the cluster 46–60 (44.5%). Finally, subjects aged more than 60 and the youngest subjects aged 15–30, are respectively 6.2 and 0.5% of the sample. For what concerns educational qualifications, descriptive analysis shows that most subjects have a high school diploma (33.5%) followed by postgraduate speciality certificate (28.2%). The rest of the sample has a middle school leaving certificate (6.3%), a bachelor's degree (13.6%) or a Master's degree (1.5%), a 5-year degree (4.4%) or a master diploma (12.6%). None among the participants has a PhD qualification. Seniority ranges from 0 to a maximum of 44 years of work in the same organization (*M* = 11.78, *SD* = 8.44).

### Items Reliability Scores

We analyzed the reliability of the items considering the Cronbach's Alphas values. The six Leadership items showed high reliability (α = 0.86), while the Cooperation component showed low values. Alpha improves after deleting three items which showed a poor adjustment in the Cooperation component, by increasing it to α = 0.71. For what concerns the Cognitive dimension, Situational Awareness and Decision-Making presented acceptable values (respectively α = 0.80 and α = 0.82). Resilience showed high reliability, while the five items of Emotion Regulation did not present acceptable values. Therefore, we removed three items and kept the items 28 and 29: the value increased up to α = 0.72. In accordance with the aim of developing a short scale, we retained 24 items that were next analyzed.

### Principal Component Analysis (PCA)

Even if the scale was designed considering six components, we could not exclude the presence of different latent factors models until the structure of the inventory was tested. Principal Component Analysis was preferred over other reduction techniques because of the heterogeneity of possible theoretical models, leaving us with a lack of prior knowledge regarding the numbers of given components and items loaded in them. Analyses were performed with a Varimax rotation to allow for component intercorrelations with a one-, two-, three- four –five, and six components, in order to determine the most interpretable solution. Based on a visual inspection of the scree plot, the five and six components solutions seemed reasonable. Where the first five components explained the 58.62% of variance, the same five-component solution has several components loadings with extremely low values (*r* < 0.30), which makes the interpretability of the dimension constrained (Russell, 2002). For such a reason we performed a parallel analysis, a Monte-Carlo simulation of eigenvalues, in order to determine the optimal number of components to retain. In the current analysis, 1000 random datasets were generated (95% CI) with the same number of cases and variables as the original dataset. According to the comparison between mean eigenvalues obtained with the observed eigenvalues from study, we kept the six-component solution over the five-component solution. Besides such methodological aspects, the six-component solution was preferred especially for theoretical reasons, since every item of the resulted components was distributed according to the six constructs. Finally, in order to obtain a more stable solution, we removed the items that showed a correlation with the component inferior to 0.60, considering that most of these also loaded moderately in the other components identified. That resulted in a reduction from five to four items for the Resilience and the Leadership components, and for what concerns the Cognitive dimension, the components Decision-Making and Situational Awareness were reduced from five to four items and from five to two items respectively. In summary, the scale is composed of a total of 18 items, based on six constructs which can present four (i.e., Leadership, Decision-Making, Resilience) or two items each (i.e., Situational Awareness, Cooperation, and Emotion Regulation). Table 2 presents the most interpretable solution.

**Table 2:** Rotated component solution of the NOTECHS+ inventory.

Item 7	833					
Item 6	800					
Item 10	770					
Item 9	766					
Item 24		802				
Item 22		780				
Item 25		780				
Item 23		736				
Item 18			842			
Item 17			820			
Item 19			734			
Item 16			684			
Item 11				899		
Item 12				870		
Item 3					880	
Item 4					844	
Item 30						878
Item 28						860

*The first component is Leadership (from item 6 to 10), the second is Resilience (from item 22 to 25), the third is Decision-Making (from item 16 to 19), the fourth Situational Awareness (items 11 and 12), the fifth Cooperation (items 3 and 4) and the last is Emotional Regulation (items 28 and 30). n, 211. Extraction Method: Principal Component Analysis. Rotation Method: Varimax. Preliminary assessment revealed a KMO value of 0.80, and Bartlett's Test of Sphericity was significant, indicating that the minimum criteria for the PCA were met. None of the off-diagonal items presented correlations > 0.90, suggesting no evidence of multicollinearity. The communality estimate resulted in an average of 0.42. Coefficient values below 0.30 were suppressed*.

### Confirmatory Component Analysis (CFA)

Confirmatory Factor Analysis (CFA) was performed on the relationships indicated in the PCA. Since there was not sufficient theoretical basis, we tested the same data for cross-validation purposes (see Fabrigar et al., 1999). We tested the validity of the solution found with the restrictions implied by the CFA (e.g., fixed cross-loadings, measurement errors, etc., see Worthington and Whittaker, 2006). Moreover, this operation was helpful to define the presence of a possible bifactor configuration, which could result in several theoretical models tested herein.

**Figure 1:**
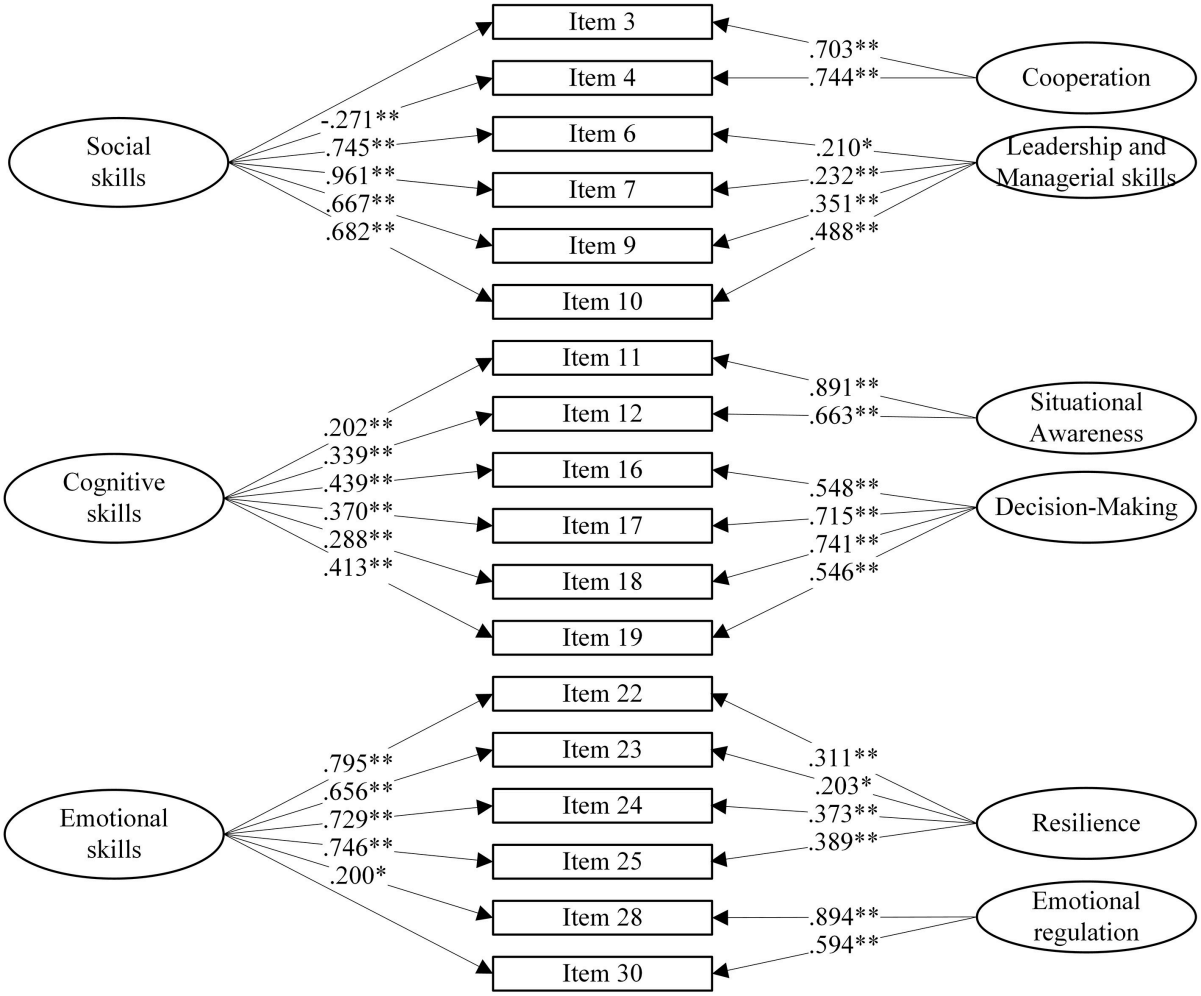
Confirmatory factor analysis of the NOTECHS+ scale. *n* = 211. ^*^p < 0.05 and ^**^p < 0.01.

The first CFA performed on the six components found and based on a single factor configuration showed a quasi-acceptable fit indexes: χ^2^_(124)_ = 195.342 (*p* < 0.001), TLI = 0.928, CFI = 0.948, RMSEA = 0.052 (Cheung and Rensvold, 2002). Considering this first results, based on several indicators (e.g., Parallel Analysis scree plot, Velicer MAP, and BIC), we investigated the presence of a possible bi-dimensional structure. The second model that we tested considered all the previous subscales together through a bifactor model to verify if they underlie a single construct (NOTECHS). The results of the bifactor analysis little improved but they were still improvable. Then, the third model focused on the presence of a single underlying construct: the NOTECHS, based on the classical four dimensions and the two new constructs comprised in a single latent factor. This resulted in an increment to an acceptable CFI value (Hu and Bentler, 1999) and in an acceptable small increment of RMSEA value [χ^2^_(106)_ = 136.486 (*p* = 0.025), TLI = 0.964, CFI = 0.978, RMSEA = 0.037]. Finally, we explored the possibility of having three possible latent dimensions based on skills configuration: Social skills (i.e., Cooperation and Leadership), Cognitive skills (i.e., Decision-Making and Situational Awareness), and Emotional skills (i.e., Resilience and Emotional Regulation), which resulted in the best fit configuration found [χ^2^_(103)_ = 108.374 (*p* = 0.339), TLI = 0.993, CFI = 0.990, RMSEA = 0.016]. In conclusion, the validated instrument is made up of 18 items referring to three dimensions (i.e., Social skills, Emotional skills, Cognitive skills) composed of six items each and the six constructs with respective items. For a graphical schema of such a model, see Figure 1.

## Discussion

Accident investigations carried out in the 70's showed that pilots' Non-Technical Skills such as Leadership, Communication, Teamwork, and Decision-making, were clearly disregarded as significant factors for safe flight operations (Cooper et al., 1980). The NOTECHS system, as stated, comprises two categories, one social and one cognitive. However, in the aviation context—and especially in the emergency context—the emotional response has a relevant role in the activities accomplishment (Beilock and Carr, 2001), since it can affect the muscular tone, breathing, cardiac, and endocrine activity. Indeed, beyond the social and cognitive categories, this study added a new dimension related to Emotional skills.

Considering the methodological approach, the mixed method used allowed for the development of a systematic and a psychometric assessment procedure. To validate this new theoretical model, we tried to verify if the six components of the NOTECHS+ scale—i.e., Cooperation, Leadership, Situational Awareness, Decision-Making, Resilience, and Emotional Regulation—could also be explained with another macro-factor; but we found no satisfying model fit-indexes. We then conceptualized another bi-factorial model that explained the four original NOTECHS as belonging to one dimension and the additional dimension as belonging to the extra Emotional skills dimension, but again indexes' values were not satisfying. Finally, we assumed and analyzed a model in which the six components were explained by three latent dimensions: Cognitive, Social, and Emotional skills. This result, is relevant because it can contribute some practical implications in relation to the assessment and the training of such competences, which could be organized following this tripartite structure. Before considering the practical aspects, below we will introduce some insights derived from the literature in support for these three dimensions coming from the confirmative analysis.

### The Social Skills: Cooperation, Leadership and Managerial Skills

Despite the fact that they belong to a unique latent dimension, the two social categories of the NOTECHS system—i.e., Cooperation, and Leadership—could seem overlapping, since both of them refer to managing group processes. However, there is a conceptual difference according to which Cooperation concerns reciprocal assistance and group climate at work (Costantini et al., 2019), whereas Leadership covers all the aspects related to initiative, coordination and goal definition (Flin et al., 2003). The studies developed by Flin (1996) on aircraft accidents showed that the best performing leaders analyse the situation, have a wide range of leadership styles and adapt their style to the situation that they experience. Among the various studies on Leadership, Zaccaro et al. (2001) reported that the leadership aspects that affect group performance are: active participation of the team leader and of all the other team members, definition of group's direction and the attempt to organize the team as to maximize team development, respect from other team members; awareness of one's own strengths and the willingness to respect the other team members and their role, encouraging open communication, including the discussion on the team's goals and on expectations about performances, which lead to commitment and consensus within the team. As a matter of fact, it was demonstrated that good leadership is important for safe performance in the workplace (Hofmann and Morgenson, 2004; Glendon et al., 2006).

For what concerns cooperation, Grice and Katz (2005) stressed the importance of cohesion in military and aviation psychology, stating that efficient teams have shared goals. Some studies found that team skills are identifiable and can be trained (Cannon-Bowers and Salas, 1998). Flin et al. (2003) showed that cooperation does not refer to job characteristics, such as quality or quantity of job outcomes, but that good cooperation originates from an open and active communication among the team members. Teamwork is achieved when the various members begin to cooperate and coordinate their efforts, by maintaining their attention focused on the task, thus mitigating interpersonal relationships dynamics inside the team (Baker and Salas, 1996). Attention to task-oriented cooperation underlines the factors that contribute to achieving the team's goals. This happens thanks to conditions such as the attribution of roles, shared mental models and feedback. These features are obtained from the skills developed in Crew Resource Management (CRM), which improves performance and reduces human error in teams, such as in aviation crews (Nullmeyer and Spiker, 2003).

### The Cognitive Skills: Decision Making and Situational Awareness

The second bi-factorial dimension regards the cognitive skills of decision-making and situational awareness, which are both relevant for sectors like emergency (Harris et al., 2016) and aviation. For what concerns the latter, Flin et al. (2010) found that most serious aviation accidents involved situational awareness issues. An analysis of the most important aviation accidents was performed between 1989 and 1992. It was found that the lack of situational awareness was the first cause of accident in 88% of the accidents related to human error (Endsley and Robertson, 2000). Situational awareness helps to manage all the information coming from the environment, through a cognitive organization, and taking into consideration human limited resources (e.g., working memory). A well-known model of situational awareness was developed by Endsley (1995) and it is made up of levels that correspond to the three elements of Situational Awareness. The first concerns the perception of the elements of the current situation; the second level refers to the interpretation of information collected; finally, the third level concerns the anticipation of future conditions. As a matter of facts, Flin et al. (2010) affirm that this predictive component of situational awareness is very important in dynamic working environments. Emergency operators shall be able to think ahead and to anticipate events. Mitchell and Flin (2008) stated that in an operational working environment there is a continuous monitoring and re-assessment of the environment as to make appropriate decisions.

Since the results of an analysis of the aviation accidents occurred between 1983 and 1987 in the United States, researchers showed that in 47% of accidents the main contributing factors were teams' poor assessment and poor Decision-Making. In the studies on aircraft pilots, the Naturalistic Decision-Making model used (i.e., a model of dynamic decision-making applicable to high-risk working environments such as aviation, see Orasanu, 1995) presents a two-stage process: assessment of the situation (Stage 1) and application of a decision-making method to choose the actions to be taken (Stage 2). Researchers noted that pilots use one or more Decision-Making processes depending on the demands of the situation, but that they also use intuition, a regulation framework and analysis of the available options. Moreover, they showed that when pilots have little time available and are faced with high risk, they use quicker strategies and apply known rules (Orasanu and Fischer, 1997). Fatigue, instead, may affect flexibility, cause an increase in the propensity to errors and affect the ability to promote an update of the situation (Harrison and Horne, 1999). Moreover, the effort required in decision-making processes rapidly depletes personal resources, thus leaving the executive function less efficient when performing other tasks (Ceschi et al., 2014, 2017a,b, 2018). As information processing increases, greater cognitive resources are required for a competent functioning. In complex environments such as HEMS, a competent decision-maker requires a variety of cognitive skills to continuously search for information to improve safety (Houghton et al., 2000). An essential point of Decision Making in NOTECHS is the structure for processing information, based on the last advance in cognitive psychology research (Flin et al., 2003).

### The Emotional Skills: Resilience and Emotional Regulation

Generally, in the aviation sector and even more in the HEMS sector, operators show levels of PTSD much higher than in the rest of the population. In particular, operators of the emergency sector show a prevalence of 14.6% on general population. Moreover, there is evidence on how the proximity, duration and intensity of the exposure to traumatic events (e.g., accidents involving children, car accidents, violent accidents) are the most significant predictors of disorders affecting front-line operators of the emergency sector (Benedek et al., 2007). As a matter of fact, exposure to critical incidents involving death or life-threatening injury is potentially an integral part of the job for emergency services personnel (Pietrantoni and Prati, 2008). In relation to the extraordinary conditions of the HEMS sector, the facts reported by Gillespie et al. (2007) show that resilience is a crucial protective factor against the development of mental disorders caused by the exposure to traumatic events of the medical personnel working in the emergency sector. Furthermore, the identification of resilience as a fundamental trait for emergency staff provided empirical evidence to what Smith and Roberts' (2003) maintained to be a protective and adaptive process. Accordingly, different disciplines focus on several aspects of resilience, resulting in diverse but interrelated definitions (Folke, 2006). Studies within the workplace have demonstrated that resilience is a significant negative mediator between the effects of job stressors and work-related psychological disorders (Bartone, 2006). Individuals with high levels of resilience adapt their coping strategies, and they even turn stressors into learning opportunities (Steinhardt and Dolbier, 2008). These studies see Resilience as a component of mediated-coping processes aiming at avoiding exhaustion through the interaction with other protective factors. The capacity to use Resilience when facing adverse events consists of a set of characteristics held by individuals. Such a conglomerate of abilities and capabilities permits the individuals to promptly direct their action, going beyond the potentially debilitating consequences of negative events. For instance, evidence shows that in burnout recovering, personal resources post-crisis growth is related to Resilience and fostered by Emotional Regulation (Fredrickson and Losada, 2005).

Furthermore, Emotional Regulation, and in particular emotional suppression, involves the necessary capacity to inhibit the first emotional reaction due to extreme situations and critical events (Mauss et al., 2007). According to Bandura (1997), stress reactions mostly depend on these coping capabilities (Prati et al., 2010) based on self-regulation processes and ability to broaden one's thoughts and actions, and they are connected to the resilience level of the individual (Ceschi et al., 2017c). Individuals with high levels of resilience adapt their coping strategies and often turn stressful factors into learning opportunities (Steinhardt and Dolbier, 2008). Moreover, through the presence of some working experiences, individuals could develop more Resilience and increase their Emotional Regulation, by managing more dynamic and intensive workplaces (Judge and Bono, 2000). In this framework, resilient individuals are those who are able to implement coping strategies, such as being focused on the problem, the capacity to take time before acting and to give and receive support from their workmates (Bartone, 2006).

## Practical Implications, Potentials, and Limitations

The NOTECHS+ system offers a systematic approach for the assessment of non-technical competences of the professionals involved in aviation and in the emergency sectors. On the whole, the indexes of the instrument are reliable for all the components analyzed. The 18-item questionnaire is characterized by brevity, which is an essential feature in such a dynamic and emergency-related context such as HEMS. Moreover, since this sector comprises both the aviation and the emergency fields, as well as technical personnel such as pilots, doctors, nurses, technicians etc., the instrument has been designed with the aim of assessing all these different profiles.

Considering some practical aspects, besides assessment, due to its brevity, the NOTECHS+ developed in this study could be used to measure the baseline and the effects of the intervention, where an intervention can be used to understand the strengths and limits of the three components considered and to then provide trainings to strengthen the non-technical components needed to deal with high-stress environments. Moreover, training could be reorganized following this tripartite skills-structure. Systematic training on NOTECHS has been provided for years now, thanks to the Crew Resource Management (CRM) training courses. Crew Resource Management courses include modules on leadership and supervision, techniques for the development of metacognitive skills, the implementation of skills on task management, recognition of critical signals, etc. (Mitchell and Flin, 2008).

As it happens for NOTECHS, Resilience and Emotional Regulation may be included in trainings and further developed with specific interventions as to understand and improve these psychological mechanisms that enable situational awareness and coping mechanisms in situations of high stress. This is especially important in fields that involve teams that endure high-stress on a daily basis. The applicability of this scale can also be extended to other teams in the healthcare system and in the military, where unforeseen events happen regularly and where it is especially important to be trained to cope effectively and maintain situational awareness under stressful conditions.

Although these results are promising, we must acknowledge several limitations that could represent the focus of future research. First, to reduce participant burden and items reliability, our indicators were limited to a small set. Second, individual differences may have had an influence on the factors found, however we did not consider personality, personal inclinations, and socio-demographic characteristics such as age or gender, education level, work experiences, etc. which might have a role in factor emergence. Third, data collected refers to cross-sectional self-reported values. Further studies could include longitudinal designs along with the inclusion of the original NOTECHS behavioral markers. Finally, the sample was composed only of Italian participants. Future research could consider possible cross-culture effects by sampling participants from different countries.

## Author Contributions

The authors discussed the contents of this article together. AnC developed the research hypotheses, devised the methodological content, and analyzed the data. ArC, VZ, EA, and RS, conferred with AnC about theoretical and empirical aspects of the study, and provided a significant contribution to the interpretation and discussion of the research findings. The final version of the manuscript was written by AnC, ArC, VZ, EA, and RS.

### Conflict of Interest Statement

The authors declare that the research was conducted in the absence of any commercial or financial relationships that could be construed as a potential conflict of interest.
